# Durability of Starch Based Biodegradable Plastics Reinforced with Manila Hemp Fibers

**DOI:** 10.3390/ma4030457

**Published:** 2011-02-25

**Authors:** Shinji Ochi

**Affiliations:** Niihama National College of Technology, 7-1 yakumo-cho, Niihama-shi, Ehime 792-8580, Japan; E-Mail: s_ochi@mec.niihama-nct.ac.jp; Tel.: +81-897-37-7742; Fax: +81-897-37-7734

**Keywords:** biodegradable composites, Manila hemp, biodegradable plastic, natural fiber, biodegradability

## Abstract

The biodegradability of Manila hemp fiber reinforced biodegradable plastics was studied for 240 days in a natural soil and 30 days in a compost soil. After biodegradability tests, weights were measured and both tensile strength tests and microscopic observation were performed to evaluate the biodegradation behavior of the composites. The results indicate that the tensile strength of the composites displays a sharp decrease for up to five days, followed by a gradual decrease. The weight loss and the reduction in tensile strength of biodegradable composite materials in the compost soil are both significantly greater than those buried in natural soil. The biodegradability of these composites is enhanced along the lower portion because this area is more easily attacked by microorganisms.

## 1. Introduction

In recent years, global concerns have included zero-emissions and increased recycling. Fiber reinforced plastics (FRP), including glass and carbon fiber reinforced plastics, have good characteristics, such as being lightweight, exhibiting high strength, and having excellent corrosion resistance. Therefore, these conventional FRP are extensively used in a wide range of fields, including automobile parts, electric products and sporting goods. However, conventional FRP impact the environment in two distinct ways. First, they are made from fossil fuels. Moreover, they are non-biodegradable, and thus exacerbate landfill shortage problems. From this perspective, the usage and disposal of conventional FRP clearly contribute to the global concerns of zero-emissions and recycling and emphasis needs to be placed on the situation involving FRP once they have been disposed.

In order to realize a sustainable society, it is essential that environmentally friendly or totally biodegradable composite materials are developed [1,2,3,4,5,6,7,8,9,10,11,12,13,14,15]. Most of the current composites comprise biodegradable plastics and a wide variety of natural plant fibers, such as flax [1,2], pineapple [3,4], bamboo [5,6,7], hemp [8,9,10], cotton [10,11], kenaf [10,12,13], ramie [14] and Manila hemp [15]. In recent years, therefore, concern has been raised regarding the fabrication of biodegradable composite materials and their subsequent mechanical properties, such as tensile strength, flexural strength, impact strength and interface strength. From a practical point of view, several studies [16,17] have examined the biodegradability of simple biodegradable plastics, while few studies have examined the biodegradability behavior and long-term durability of the biodegradable composite materials using natural fiber bundles. Evaluating not only the mechanical properties of biodegradable composite materials but also their degradability in soil or compost is equally as important.

The purpose of the present study is to evaluate the biodegradability of biodegradable composite materials and their time-dependent mechanical properties. Specimens of biodegradable composite materials were fabricated from Manila hemp fiber bundles for reinforcement and starch-based biodegradable plastics as a matrix. These specimens were exposed for 240 days to a natural soil and 30 days to a compost soil generated using a garbage-processing machine. After the biodegradability tests, weights were measured and both tensile strength tests and microscopic observation were performed to evaluate the biodegradation behavior, weight loss and decrease in strength of the biodegradable composite materials.

## 2. Experimental Procedures

### 2.1. Materials

Manila hemp fiber reinforced biodegradable plastics (MHFRP) were made from Manila hemp fiber bundles for the reinforcement and starch-based emulsion-type biodegradable plastics (CP300; Miyoshi Oil & Fat Co., Ltd., Japan) as the matrix. This plastic contains fine particles (4.6 μm diameter) suspended in an aqueous solution with a mass content of approximately 40%. Long hemp fiber bundles (100 to 200 μm diameter; 200 mm length) without any surface treatment, were used in the present study.

### 2.2. Specimen molding method

**Figure 1:**
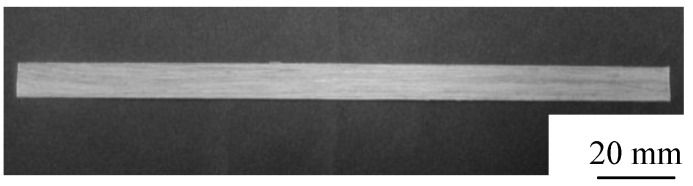
Survey photograph of Manila hemp fiber reinforced biodegradable plastics (MHFRP) specimen.

At first, prepregs were produced by placing the emulsion-type biodegradable resin on the surface of the Manila hemp fiber bundles. These prepregs were then dried at 105 °C for 7.2 ks in an oven. Next, MHFRP specimens were compression molded in a hot-press machine. The prepregs were set in a metallic mold, and then heated to 130 °C. The metallic mold was held at 130 °C for 0.3 ks, and the specimens were subsequently hot-pressed at 130 °C and at 10 MPa for 0.6 ks. The dimensions of the fabricated unidirectional long fiber reinforced composite materials are 10 mm × 200 mm × 1 mm. The fiber content of all specimens is fixed to be 50% by weight. A photograph of a fabricated MHFRP specimen is shown in Figure 1.

### 2.3. Biodegradability tests

**Figure 2:**
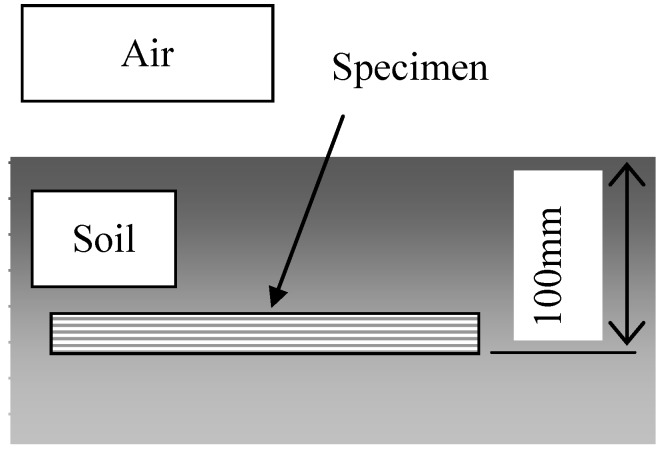
Schematic illustration of the biodegradable test (Condition 1).

**Figure 3:**
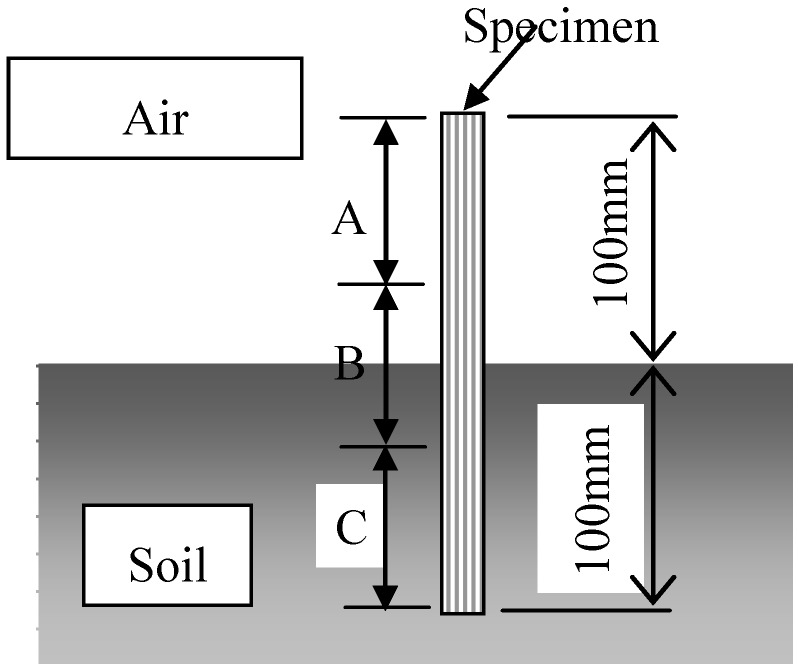
Schematic illustration of the biodegradable test (Condition 2).

**Figure 4:**
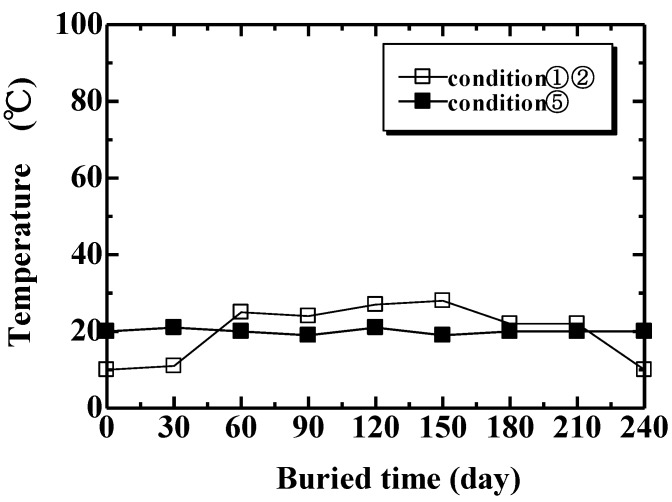
Temperature of natural soil (condition 1, 2) and room (condition 5).

**Figure 5:**
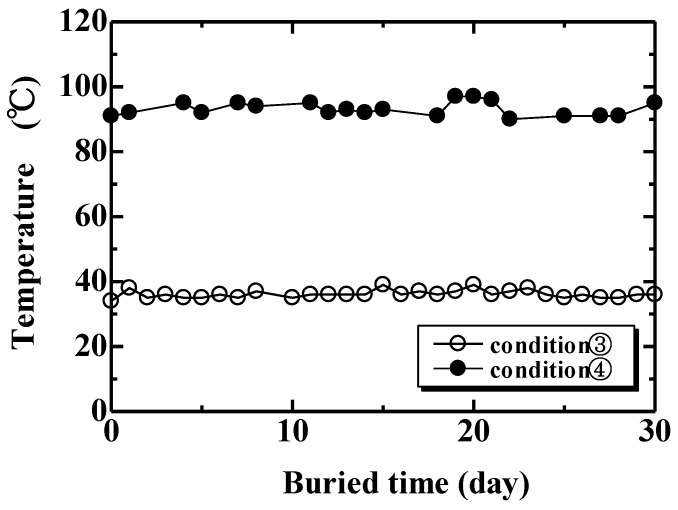
Temperature of compost soil (condition 3,4).

Biodegradability tests were carried out under five conditions. In Condition 1, specimens were completely buried in a natural soil at a depth of 100 mm, as shown in Figure 2. In Condition 2, a 100 mm section of the specimens were buried in natural soil as shown in Figure 3. This examination under Condition 2 was to observe the biodegradability from three factors: air (Figure 3 A), air and soil (Figure 3 B) and natural soil (Figure 3 C). Conditions 3 and 4 examined specimens in compost soils generated using two types of garbage-processing machines (BDG-150 and BG-CX20; Hitachi Ltd.). The BDG-150 operated at around 36 °C (Condition 3), and the BG-CX20 operated at around 90 °C (Condition 4). After a trial operation for several days in order to activate microorganisms in the compost media (wood chips), MHFRP samples were placed within the processing media, and the biodegradation behavior of the composites was investigated. Specifically, samples were placed inside a nylon netting to aid in recovering the degraded samples from the media, and then the netting and sample were buried in the compost media according to the experimental conditions. In Condition 5, specimens were kept indoors, in desiccators, at 20 °C and 50% relative humidity. The duration of the biodegradability tests was 240 days for Conditions 1, 2 and 5, and 30 days for Conditions 3 and 4. The temperatures of the examining environments of Conditions 1, 2 and 5 (20 °C) as well as Conditions 3 (36 °C) and 4 (90 °C) are shown in Figure 4 and Figure 5, respectively.

### 2.4. Evaluation of biodegradability

After biodegradability tests, any materials attached to the surface of the specimens were removed by washing in running water. Microscopy observations and analysis of both weight loss and tensile strength were carried out after drying the samples at 50 °C for 24 hours.

The weight loss of composted samples was evaluated using the following equation:

Weight loss [%] = (W_0_ – W_1_) / W_0_ × 100 (1)

where W_0_ and W_1_ are sample weights before and after biodegradability test, respectively.

Tensile tests for MHFRP were carried out on five prepared specimens using an Instron Testing Machine (Model 4482). The cross sectional areas were calculated from width and thickness of the specimen measured using vernier calipers after decomposition tests. Aluminum tabs of 2 mm thickness were glued at the both ends of the tensile specimen to prevent damage from being introduced by gripping of the specimen in the testing machine. The tensile strength tests were performed at a strain rate of 0.16/min and a gauge length of 30 mm. After biodegradability tests, the microscopic characteristics of the samples were examined using scanning electron microscopy (SEM).

## 3. Results and Discussions

### 3.1. Mechanical properties

**Figure 6:**
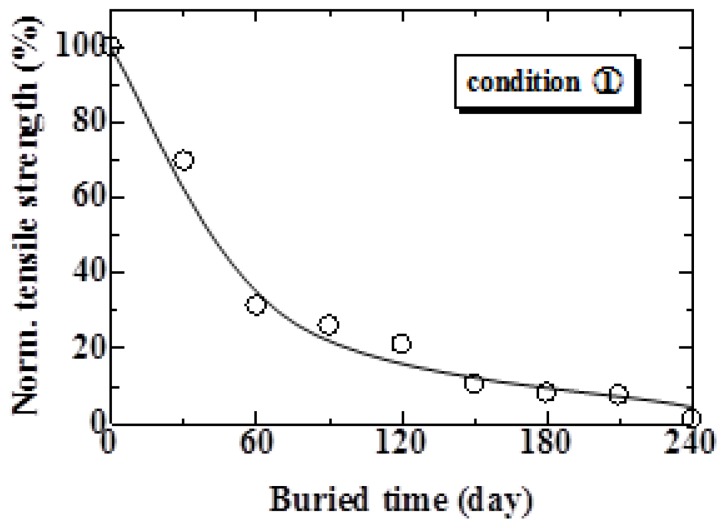
Relationship between normalized tensile strength and burial period under Condition 1.

**Figure 7:**
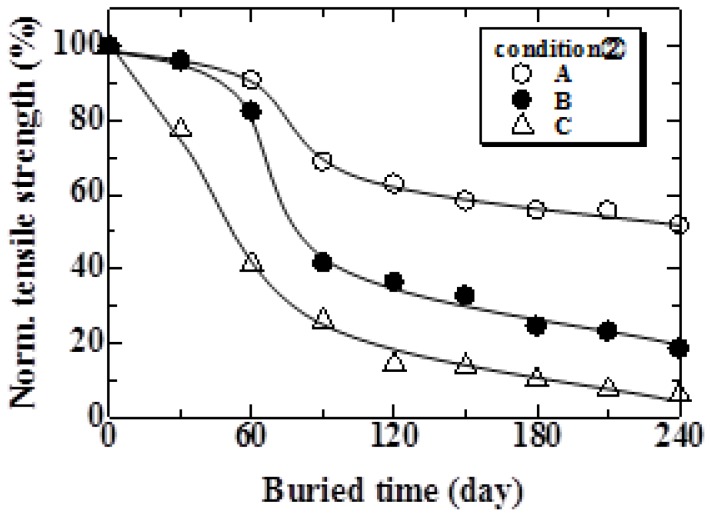
Relationship between normalized tensile strength and burial period under Condition 2.

The relationship between the tensile strength of the MHFRP and burial time for Condition 1 is shown in Figure 6. The tensile strength rapidly decreases during the initial 1 to 60 days. After 60 days, the tensile strength gradually decreased to 0 MPa. The tensile strength of MHFRP under Condition 2 is shown in Figure 7. The tensile strength of each part of the MHFRP (A, B and C in Figure 3) indicates a sharp decrease in the early stages, followed by a gradual decrease. More specifically, the tensile strength of parts A and B rapidly decreased from 60 to 90 days, and after 90 days, slowly decreased. On the other hand, the tensile strength of part C decreased rapidly up to 30 days, and decreased gradually after 90 days. The tensile strength decreased largest in the part buried in soil (Figure 3C). Moreover, the tensile strength of part C under Condition 2 shows a tendency that resembles that seen in Condition 1 (Figure 6). This is because the specimen in Condition 1 and part C in Condition 2 are both completely buried in natural soil.

**Figure 8:**
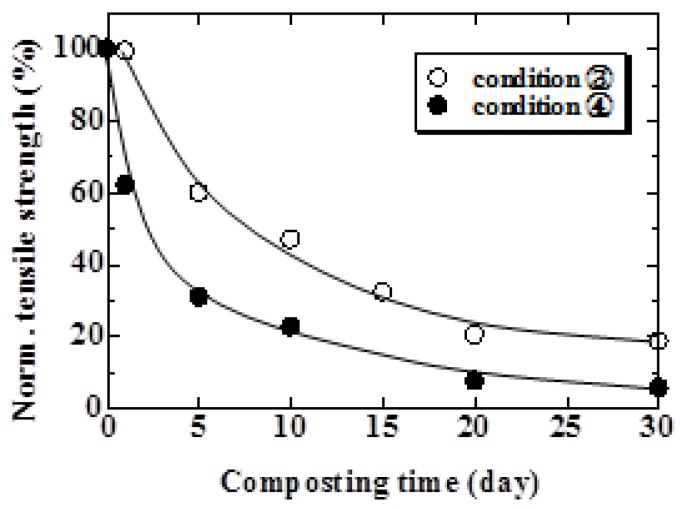
Relationship between normalized tensile strength and composting time under Condition 3 and 4.

The relationship between normalized tensile strength and composting time for the conditions using the compost soil is shown in Figure 8. As seen in this figure, the tensile strength of the specimen under Conditions 3 and 4 decreases after a composting test of 5 days and 1 day, respectively. The period of decreasing tensile strength compared to that with natural soil (Figure 6 and Figure 7) is remarkably short. Moreover, the tensile strength of the composted specimen at high temperature showed the largest decrease; the strength gradually decreased to the 0 MPa level at 30 days.

The tensile strength of the MHFRP in Condition 5 did not decrease. Together, these results suggest that the order of the extent of the decrease in tensile strength was, from greatest to smallest; Condition 4 (compost soil, 90 °C), Condition 3 (compost soil, 36 °C), Condition 1 (natural soil), Condition 2 C (natural soil), Condition 2 B (natural soil and air), Condition 2 A (air), with Condition 5 (room) resulting in no decrease in tensile strength.

### 3.2. Biodegradation behavior

**Figure 9:**
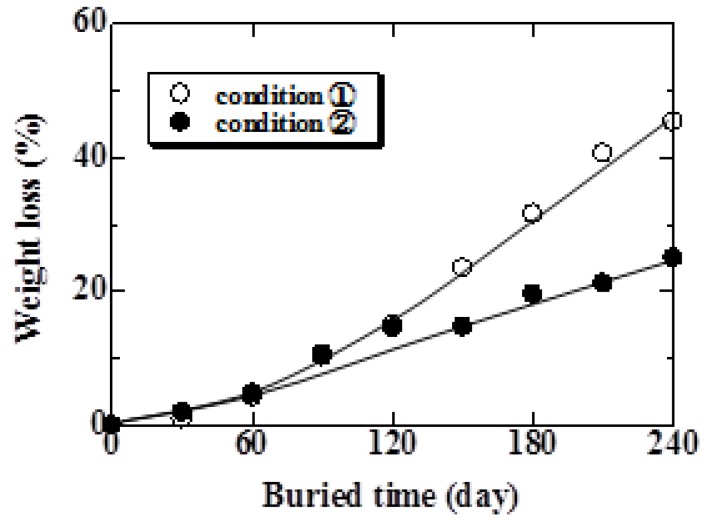
Relationship between weight loss and burial period for Conditions 1 and 2.

**Figure 10:**
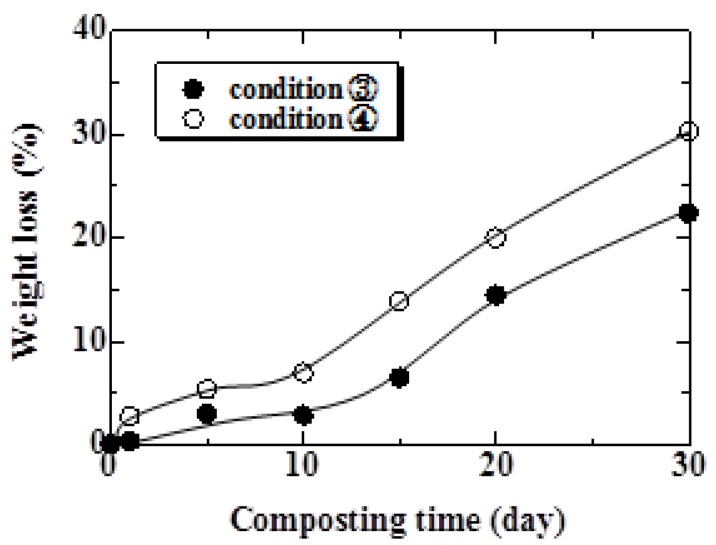
Relationship between weight loss and composting time under Conditions 3 and 4.

The relationship between weight loss and burial time for Conditions 1 and 2 are shown in Figure 9. As shown in this figure, weight loss increased with increasing burial time. Comparing Condition 1 with Condition 2, the weight loss of the specimen examined under Condition 1 was approximately 1.8 times of that under Condition 2 at 240 days. These results suggest that the specimen with many parts contacting microbes in the soil biodegrade early. The relationship between weight loss and composting time is shown in Figure 10. The weight loss under Conditions 3 and 4 was 23 and 30% at 30 days, respectively. As shown in this figure, in both conditions, weight loss increased gradually for 10 days and then suddenly increased thereafter. From the data in Figure 8, tensile strength decreased rapidly until 10 days and then gradually decreased thereafter. The strength of hemp fiber is influenced by the degree of polymerization of the cellulose [18]. This behavior suggests that the degree of polymerization of the cellulose decreased, and fiber itself biodegraded thereafter.

In the case of Condition 1, the weight loss exceeded 20% at 150 days. In the case of Condition 4, this amount of weight loss was seen at 20 days. Moreover, weight loss did not occur in specimens under Condition 5. These results clearly indicate that the atmosphere influences the biodegradability of the MHFRP greatly. The MHFRP treated at a high temperature in the compost soil biodegraded the fastest.

### 3.3. Microscopic observations

**Figure 11:**
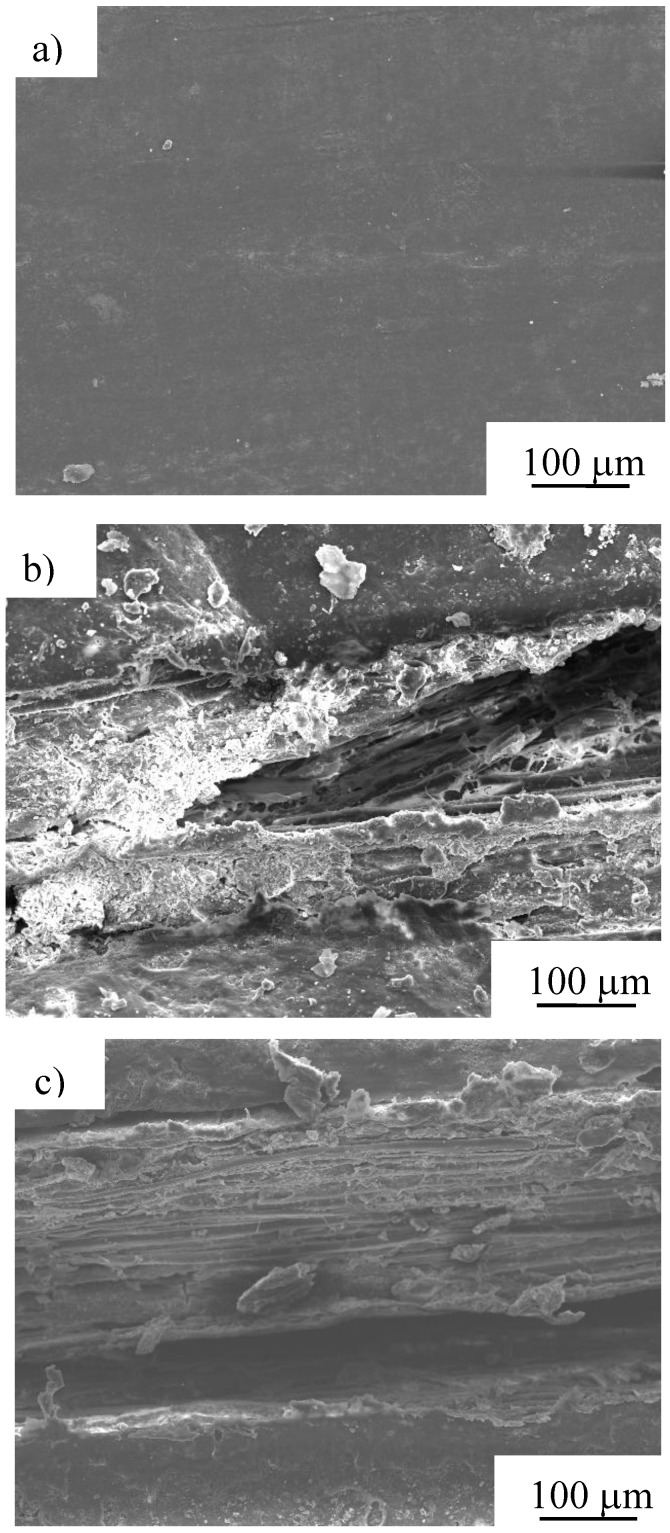
SEM photomicrographs of after biodegradable test of MHFRP after **a)** 30, **b)** 180 and **c)** 240 days burial (under Condition 1).

Scanning electron microscopy (SEM) photomicrographs of the specimen tested under Condition 1 are shown in Figure 11. The specimens depicted in Figure 11 a, b and c were tested after burial times of 30, 180 and 240 days, respectively. As shown in these figures, the change in condition of the specimen is remarkable with increasing burial time. Only the surface resin can be seen on the specimen at 30 days. At 180 days, the surface resin of the specimen has degraded, exposing the internal fibers. At 240 days, delamination is seen between the fibers and the resin, and single fibers assembling fiber bundle were observed.

**Figure 12:**
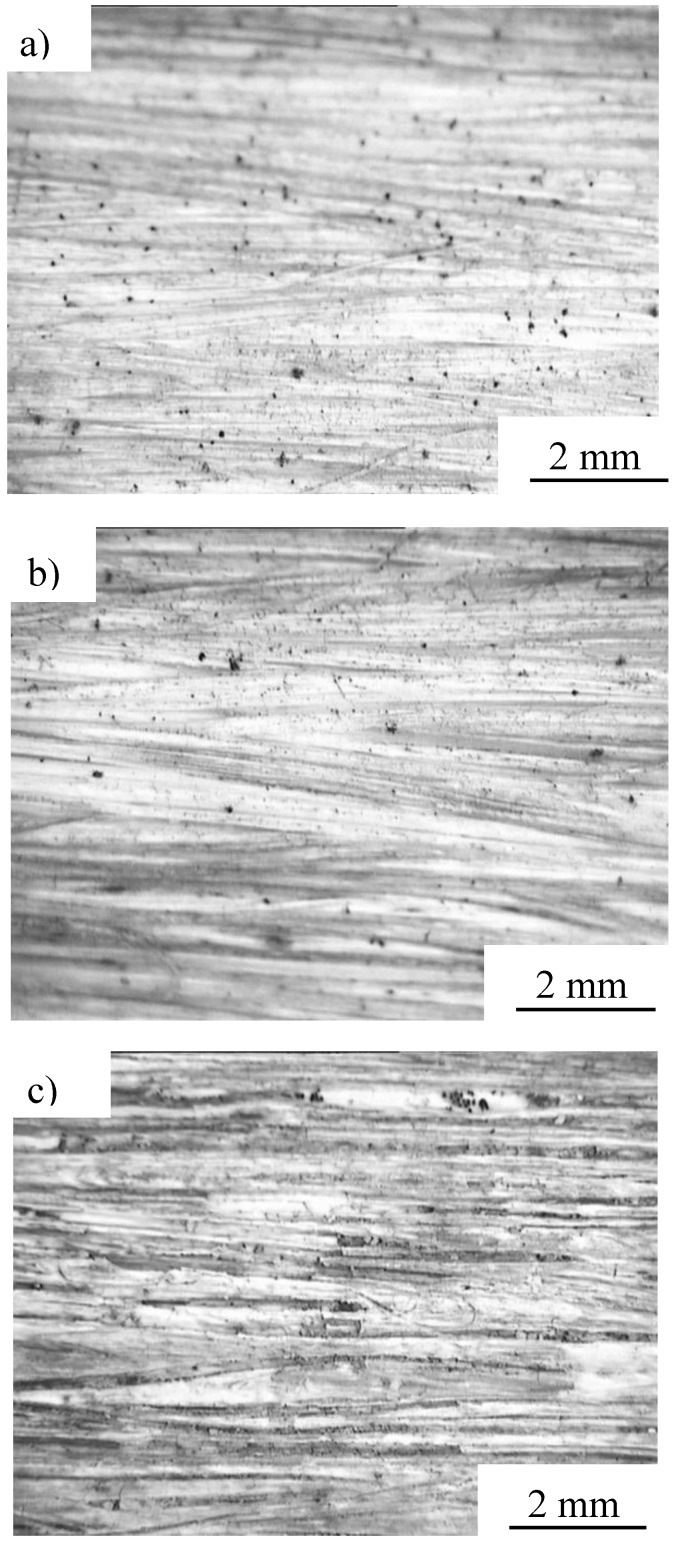
Survey photomicrographs of MHFRP tested after 240 days. **a)** upper, **b)** middle and **c)** lower part of the specimen (air; air and natural soil; natural soil exposed, respectively) under Condition 2.

Survey photomicrographs of the specimen biodegraded for 240 days in the case of Condition 2 are shown in Figure 12. The upper, middle and lower parts of the specimen (air; air and natural soil; natural soil, respectively) are depicted in Figure 12 a, b and c, respectively. From these figures, changes in the condition of the upper part of the specimen are not observed, however, at the lower part, whitening of the resin and exposure of the fiber bundle was seen. The change in state of the specimen seen here influences the tensile strength (see Figure 7). MHFRP show remarkable biodegradation when in contact with microbes in the soil.

**Figure 13:**
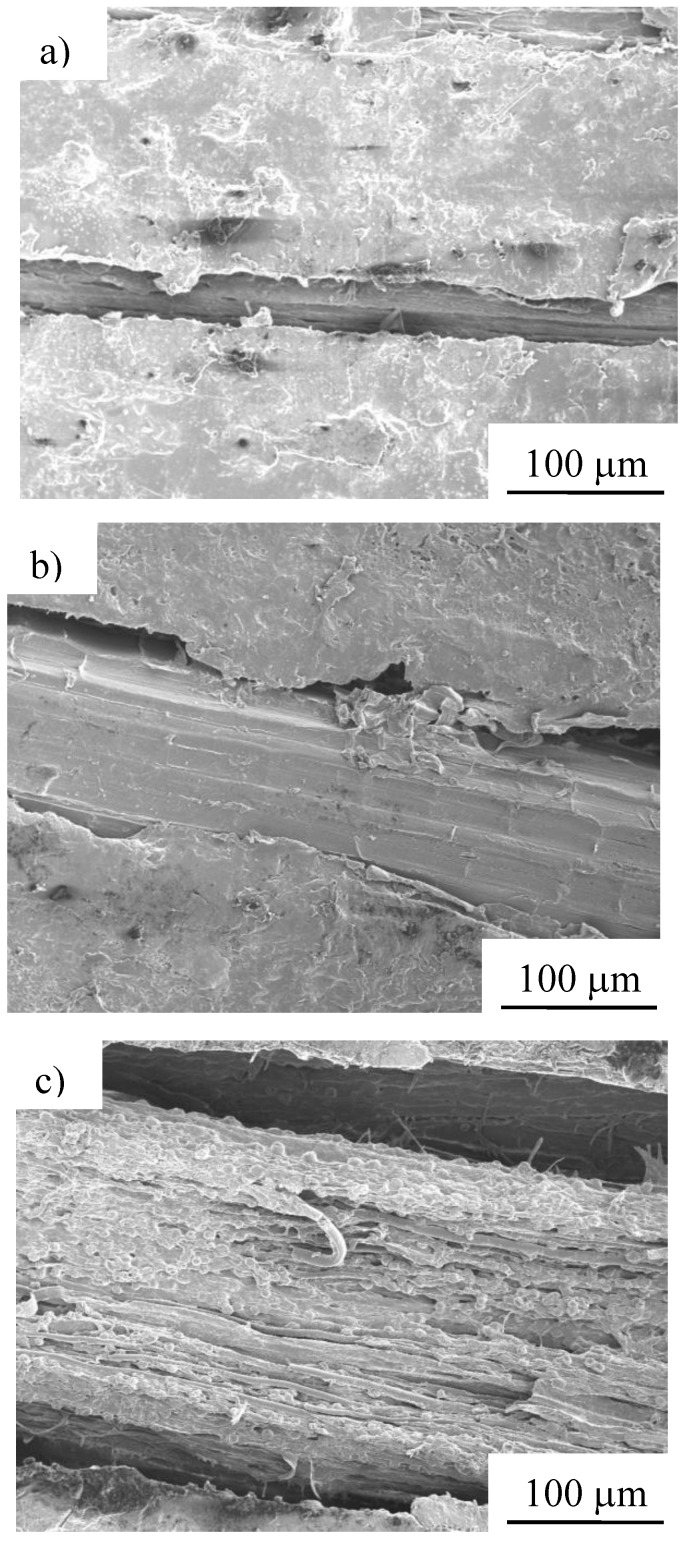
SEM photomicrographs of after composting test of MHFRP specimens after: **a)** 5, **b)** 10 and **c)** 30 days (under Condition 3).

SEM photomicrographs of the specimen tested under Condition 3 are shown in Figure 13. From these figures, it can be observed that the fiber bundle is exposed in only 5 days, delamination between the fiber and resin is seen after 10 days, and the fiber bundle disintegrates to single fibers in 30 days.

These results indicate that biodegradability was hastened compared in natural environments by processing it with compost. In the case of Condition 5, change of the state of the specimen did not occur. In the atmosphere, there are few changes of state, and underground, exposure of the fiber was observed. In the compost, the state of the specimens treated at high temperature changed remarkably. Moreover, the decomposition sequence for MHFRP can be described as follows: (i) the biodegradable resin at the specimen surface decomposes, (ii) Manila hemp fiber bundles embedded in the resin are exposed, (iii) the biodegradable resin in the vicinity of the fiber-resin interface is decomposed, resulting in interfacial gap formation, (iv) decomposition of both fibers and biodegradable resin progresses remarkably.

## 4. Conclusions

The biodegradability and long-term durability of Manila hemp fiber reinforced biodegradable plastics was studied for 240 days in natural soil and 30 days in compost soil. After biodegradability tests, weights were measured and both tensile strength tests and microscopic observation were performed to evaluate the biodegradation behavior of the developed composites. The results obtained are summarized as follows:

(1) In the case of composting, the tensile strength of Manila hemp fiber reinforced composites decreased 80% after 20 days. In the case of a natural atmosphere, the tensile strength of specimens in soil decreased 80% after 90 days. Furthermore, differences of condition could be seen between parts of a differentially exposed specimen; strength decreased for the part under soil more so than the part exposed to air.
(2) Weight loss of the specimen completely buried under the soil showed a value of approximately 1.8 times that of the specimen of which only half was buried under the soil at 240 days (45% *vs.* 25%, respectively). A weight loss of 30% was observed with the specimen in compost at 90 °C for 30 days and a similar level of weight loss was seen in natural environments at 180 days.
(3) The state of the parts of the specimens in soil changed remarkably. In the atmosphere, biodegradation is negligible. On the other hand, when the specimen interacts with microbes in either the natural or compost soil, weight, tensile strength and state of the specimen change remarkably.
(4) The decomposition sequence for MHFRP can be described as follows. The biodegradable resin at the specimen surface first decomposes, followed by exposure of the Manila hemp fiber bundles embedded within the resin. Next, the biodegradable resin in the vicinity of the fiber-resin interface decomposes, resulting in the formation of interfacial gaps. Finally, substantial decomposition of both the fibers and biodegradable resin occurs.
